# Synthetic biology: biology by design

**DOI:** 10.1099/mic.0.069724-0

**Published:** 2013-07

**Authors:** M. A. J. Roberts, R. M. Cranenburgh, M. P. Stevens, P. C. F. Oyston

**Affiliations:** 1 Barts and The London School of Medicine and Dentistry, Queen Mary University of London, Garrod Building, Turner St., Whitechapel, London E1 2AD, UK; 2 Prokarium Ltd, Stephenson Building, Keele Science Park, Keele, Staffordshire ST5 5SP, UK; 3 The Roslin Institute & Royal (Dick) School of Veterinary Studies, University of Edinburgh, Easter Bush, Midlothian EH25 9RG, UK; 4 Biomedical Sciences, DSTL Porton Down, Salisbury, Wiltshire SP4 0JQ, UK

Synthetic biology can be defined as the design and construction of novel biologically based parts, devices and systems, as well as the redesign of existing natural biological systems, for useful purposes. It builds on genetic engineering, being design-driven genetic engineering encompassing engineering concepts of standardization and abstraction (Endy, 2005). One of the technical advances that has significantly increased the ability to undertake synthetic biology has been to artificially synthesize DNA, and thus create DNA parts. So far, the peak achievement has been the synthesis and assembly of a small bacterial genome which was transferred to a bacterial cell devoid of DNA to create a novel replicating micro-organism (Gibson *et al.*, 2010). A great diversity of synthetic biology applications exists, many in the early research phase, which include using microbes as biofactories or as biological computers (Bonnet *et al.*, 2012; Oldham *et al.*, 2012). In this issue of *Microbiology* we have assembled a collection of papers to showcase the current state of synthetic biology research, and to convey the potential impact of synthetic biology on biological sciences.

The synthetic biology field itself is diverse but can broadly be divided into two main themes: bottom-up approaches creating truly artificial life *de novo*, and top-down approaches to design systems based on known biology to perform a specific task. The latter can involve designing metabolic and signalling pathways inside cells to achieve a specific purpose. Within this top-down design, biological elements (promoters, gene products, etc.) can be thought of as parts being assembled into a system. The top-down approach has the advantage of using the host cell (termed the chassis) and being able to make use of the co-factors, metabolites, transcription pathways and other components that it already possesses, but does have the potential disadvantage of potential crosstalk between the endogenous systems present in the chassis and the introduced synthetic systems (Saito, 2010; Verhamme *et al.*, 2002). The papers within this issue focus on the top-down approach.

As the aim of synthetic biology is to design a system to achieve a required outcome, researchers rely heavily on *in silico* modelling and whole-system analysis (’omic analysis) to provide data about the effect of perturbations, allowing parts encoded in DNA to be characterized and optimized. The data provide the basis to bring component parts together in different combinations to produce predictable devices with the outcomes predicted through modelling. An example of developing these standard parts is given in the Bartosiak-Jentys *et al.* (2013) paper, which describes the creation of a modular system for the design of *Geobacillus* and defins the parts that are created. The authors also describe how this may be applied in the design of improved bioethanol-producing strains. The parts themselves can be specifically modified to alter the desired outcome. The review in this issue by Arpino *et al.* (2013) describes these design parameters and how they can be modified in both prokaryote and eukaryote microbial systems. For example, different ribosome-binding sites can alter protein copy number, resulting in different outcomes from the synthetic system. Once defined, there is the ability to add these parts together to produce a system with a predictable defined output. A nice example of how using a small range of defined parts can be used to generate complex outcomes is presented by Chang *et al.* (2013). Here the authors use a simple bacterial two-component system to produce a range of outputs through careful variation of a phosphatase. This paper also highlights the utility of model-based design. Such technology has huge potential applications in industry. Being able to synthesize products in a biologically controlled way opens up new methods of manufacture, as highlighted in the Donald *et al.* (2013) paper. In this work the authors describe how expression can be optimized using synthetic biological approaches to modify the chassis, in this case to produce a vaccine.

Synthetic biology has been identified as a technology that has huge potential to transform the way we work, and this step change in our use of biology has been recognized not just in the scientific community but in the wider social sphere as well. As a result, a number of governments have been shaping policy and developing science funding to specifically support synthetic biology (Pei *et al.*, 2012; Zhang, 2011). In particular, how does current international legislation apply to synthetic biology (Bubela *et al.*, 2012)? In the UK, for example, a cross-government group (The Synthetic Biology Roadmap Coordination Group, 2012) developed the Synthetic Biology Roadmap to bring together all the different interested parties and communities and to identify what government support is needed to develop this science within the UK, both for pure understanding and to drive translational research. The Roadmap also considered approaches to the ethical and legal issues. These latter areas have been raised as a matter of concern in a number of countries, and highlighted recently in the US (Roehr, 2010), reflecting the ethical, safety and regulatory considerations that apply to any new technology but, given the potential for self-replication, have particular significance in synthetic biology. With the emphasis on making manipulation of biology easier, synthetic biology also raises significant implications of dual use of synthetic biology for nefarious purposes, which also need consideration in ethical, legal and regulatory contexts (Samuel *et al.*, 2009). Such considerations have also been the basis of activity within national learned academies, culminating in the six academies symposia between the science and engineering academies of the UK, China and the US. These meetings resulted in opinion pieces regarding ways of progressing synthetic biology research for the benefit of humanity while avoiding the potential pitfalls (OECD & The Royal Society, 2011). These issues will become especially important if we consider the possible environmental release of biological devices. Developing methods to contain and control the biological devices that we produce is thus a significant area of research. A review article in this issue by Wright *et al.* (2013) looks at current research in this area, and how scientific solutions can give us control over the spread of the synthetic systems we design.

As indicated above, synthetic biology is a priority area for funding in a number of countries. This is now evolving into an internationally structured area, with larger international research networks being established, such as the EraSynBio network between funding bodies in both Europe and the US. Just as the technology requires a multi-disciplinary effort, so the science requires an international approach and frameworks. If, for example, there are to be standardized biological parts, such as using the Biobricks standard (Canton *et al.*, 2008), researchers will have to work together, and within their domestic regulations, to achieve that.

The articles published in this issue highlight the promise and hurdles that synthetic biology must overcome to produce the future designer microbes that could transform our world. Quite what that future will be is left for the reader to imagine, but there can be no doubt synthetic biology will play an important role.
